# Integrated Molecular Characterization of HER2-Low Breast Cancer Using Next Generation Sequencing (NGS)

**DOI:** 10.3390/biomedicines11123164

**Published:** 2023-11-28

**Authors:** Jean-Louis Merlin, Marie Husson, Nassim Sahki, Pauline Gilson, Vincent Massard, Alexandre Harlé, Agnès Leroux

**Affiliations:** 1 Biopathology Department, Institut de Cancérologie de Lorraine—Alexis Vautrin, CNRS UMR7039 CRAN Université de Lorraine, 6 Avenue de Bourgogne, 54519 Vandœuvre-lès-Nancy, France; 2 Methodology Biostatistics Unit, Institut de Cancérologie de Lorraine—Alexis Vautrin, 54519 Vandoeuvre-lès-Nancy, France; 3 Medical Oncology Department, Institut de Cancérologie de Lorraine—Alexis Vautrin, 54519 Vandœuvre-lès-Nancy, France

**Keywords:** Breast cancer, HER2-low, next generation sequencing, copy number variation, antibody-drug conjugate, molecular diagnosis

## Abstract

Based on immunohistochemistry (IHC) and in situ hybridization (ISH), HER2-low breast cancers (BC) subtype—defined as IHC1+ or IHC2+/ISH− tumors—emerged and represent more than half of all BC. We evaluated the performance of NGS for integrated molecular characterization of HER2-low BC, including identification of actionable molecular targets, copy number variation (CNV), and microsatellite instability (MSI) analysis. Thirty-one BC specimens (11 HER2+, 10 HER2−, and 10 HER2-low) were routinely analyzed using IHC and ISH, and were selected and analyzed using NGS for gene mutations including *ESR1*, *PIK3CA*, *AKT1*, *ERBB2*, *TP53*, *BRCA1*, and *BRCA2*, CNV, and MSI. CNV values for the *ERBB2* gene were significantly (*p* < 0.001) different between HER2+, and either HER2-low or HER2− tumors with mean values of 7.8 (SD = 6.8), 1.9 (SD = 0.3), and 2.0 (SD = 0.3), respectively. Using 3.25 as the cutoff value, 96.8% overall concordance of HER2 status was achieved between IHC and NGS compared to IHC and ISH. Using NGS, gene mutations and amplifications were detected in 68% (21/31) and 19% (6/31) of the cases, respectively. One case of MSI was detected in a HER2-negative and ISH unamplified case. Beside IHC, NGS allows the identification of HER2-low subtype simultaneously, with the detection of multiple actionable gene mutations being helpful for molecular board treatment selection.

## 1. Introduction

Together with hormone receptors (HR) status, based on estrogen (ER) and progesterone receptor (PR) expression, HER2 status is standard for the diagnosis of breast cancer (BC). It is evaluated using immunohistochemistry (IHC), and the expression of HER2 protein, encoded by *ERBB2* gene, is scored on a scale of 0 to 3+ depending on staining intensity and the percentage of tumor cells positive for HER2 [1]. In cases with intermediate overexpression (HER2 2+) obtained by IHC, in situ hybridization (ISH) is used to detect the amplification of the *HER2* gene using fluorescent or chromogenic probes which bind to the *ERBB2* gene. As a reference, labeling of chromosome 17 centromere (CEP17) is performed and tumors are declared ISH positive when a high level of gene amplification is detected (ERBB2/CEP17 ratio > 2.0).

**Figure 1:**
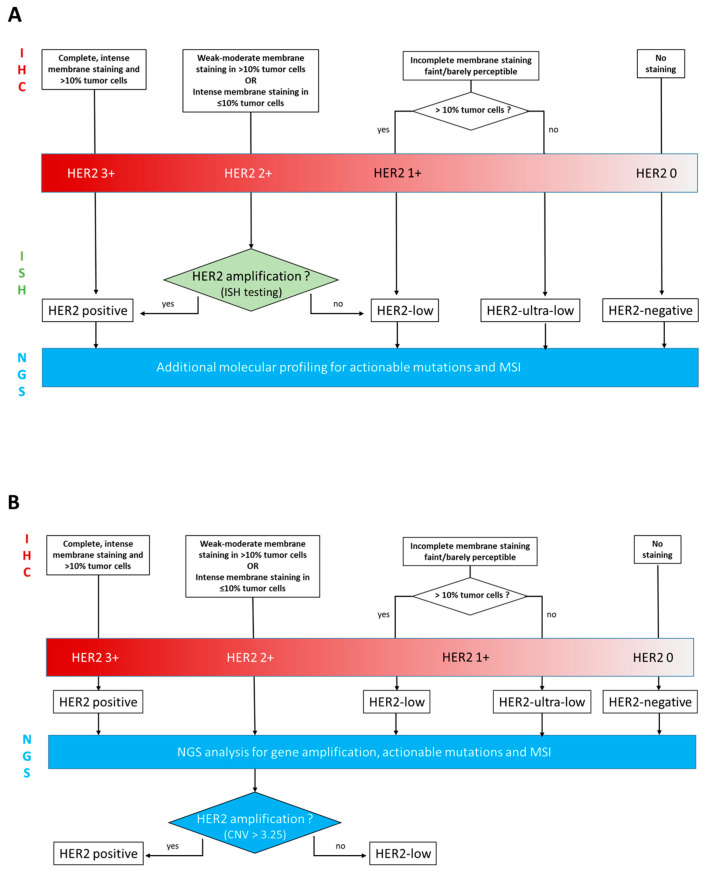
Workflow diagrams illustrating the aim of this study: (**A**) routine workflow including IHC +/− ISH +/− NGS; (**B**) experimental workflow including IHC and NGS.

As illustrated by Figure 1, HER2-positive status is based on strong (3+) or intermediate (2+) IHC overexpression with gene amplification (ISH+). Ten to 20% of BC are HER2-positive [2,3], and patients with HER2-positive BC are eligible for targeted therapies directed against HER2 protein; either monoclonal antibodies (MAbs) such as trastuzumab and pertuzumab, or kinase inhibitors such as lapatinib and, more recently, antibody–drug conjugates (ADC) such as trastuzumab deruxtecan.

There is growing interest in understanding the importance of low HER2 expression in BC, defined as IHC1+ or 2+ and ISH-negative (HER2/CEP17 ratio < 2.0), as recently highlighted in the last update of the guidelines of the American Society of Clinical Oncology/College of American Pathologists (ASCO/CAP) [4].

However, identification of HER2-low BC can be equivocal. Recently, the results of a multi-institutional assessment of pathologists scoring HER2 using immunohistochemistry were reported. Using the 4-category HER2 IHC scoring system [5], substantial discordance within the intermediate categories (IHC1+ and IHC2+) was observed with less than 1% and 3.6% agreement, respectively. Within the IHC score of 0 cases, the discordance was also substantial, with an overall agreement of only 25%. Adjustment of the criteria has been proposed to reduce the interobserver variability, suggesting that the modified 2007 ASCO/CAP criteria were more reproducible in distinguishing HER2-negative from HER2-low cases than the 2018 ASCO/CAP criteria [6]. Even in such conditions, the reproducibility was not fully adequate and was not improved by adding ISH. Since the threshold between HER2 IHC 0 and 1+ is now becoming clinically actionable, this could lead to a suboptimal identification of patients eligible for HER2-low targeted therapies. Since differentiating between the HER2 IHC score 0 and 1 + has not been clinically significant until recently, the updated recommendations for the standardized pathology report for HER2 testing were published in compliance with 2023 ASCO/CAP updates and 2023 ESMO consensus statements [7]. These recommendations mainly include the adoption of standardized procedures and guidelines, the promotion of specialized training for pathologists in interpreting HER2 expression, specifically in the lower expression level, and the development of artificial intelligence-based procedures.

The rate of HER2-low BC is about 40–65% of HR+ tumors, and 23–40% of HR-negative tumors express low levels of HER2 [8]. HER2-low cancers are reported to be more frequently observed in clinical stages I–II, ER+, ductal, histologic grade 1 or 2, and luminal molecular subtypes BC. When compared to HER2− (IHC 0) and HER2+ (IHC3+ or IHC2+ ISH amplified) BC, HER2-low BC had lower Ki-67 and higher ER positivity [9,10]. In a recent paper [11] examining 12,965 BC patients, among which 635 were stage IV patients, HER2-low BC represented more than half of both cohorts (59% overall, 53% stage IV). HER2-low BC were more frequently HR-positive than IHC 0 BC. Further, no survival differences were found between HER2-low and IHC 0 among stage IV patients.

HER2 expression is dynamic, with a higher conversion rate from HER2-low to HER2− than the reverse, and discordant between primary tumors and advanced disease [12]. These results highlight that, when feasible and safe, it is useful to obtain repeated biopsies at the time of disease progression, as this may affect treatment options.

The first HER2-targeting therapy that was widely studied for HER2-low disease was trastuzumab. The NSABP B-47 trial, a randomized phase 3 trial including 3270 women with high-risk and HER2-low BC comparing chemotherapy with or without trastuzumab [13], showed no difference in invasive disease-free survival between the groups. Later, a phase 2 study showed little benefit with pertuzumab [14] for the treatment of patients with HER2-low BC. Based on these results, the use of anti-HER2 remained limited to patients with HER2+ tumors, until the development of HER2-directed antibody–drug conjugates (ADC-HER2) such as trastuzumab deruxtecan (T-DXd).

ADC-HER2s consist of a monoclonal antibody that delivers a chemotherapy molecule directly to HER2-expressing tumor cells, resulting in the targeted killing of cancer cells while minimizing damage to healthy cells [15]. Historically, the first ADC-HER2 put on the market was trastuzumab emtansine or TDM-1, whose interest in HER2+ breast cancers has been widely demonstrated whether in metastatic [16,17,18], adjuvant [19,20], or neoadjuvant [21] settings.

Trastuzumab deruxtecan (T-DXd) is an ADC composed of a HER2-targeting monoclonal antibody, a cleavable ligand. The cytotoxic payload is deruxtecan, a potent topoisomerase 1 inhibitor. T-DXd has a high drug-to-antibody ratio of 8:1 [22]. Results from phase II [23] and phase III [24] clinical trials have shown the efficacy of T-DXd in patients with metastatic HER2+ breast cancer, with a higher response rate, longer PFS, and higher survival rate at 1 year than TDM-1 [25].

Recently, the results of the phase 2 DAISY trial [26], evaluating the efficacy of T-DXd in patients with HER2+, HER2-low, and HER2- metastatic BC revealed that, although T-DXd efficacy increased when HER2 expression was high, a mild activity was also observed in patients with HER2− tumors. This trial highlighted that although HER2 expression is a determinant of T-DXd efficacy, very low levels of HER2 could drive T-DXd cellular uptake and/or other mechanisms independent of HER2, which may also be partially involved.

Beside HR and HER2 status, additional molecular targets are emerging in BC, revealing the need for extensive molecular diagnosis to envisage precision therapy. ESMO scale for actionability of molecular targets (ESCAT) ranges 6 classes based on the level of evidence currently available (Table 1), and the clinical indication of actionability in a specific cancer type to assist oncologists in making a therapeutic decision [27]. In this scale, the genomic alterations are ranked according to their clinical benefit for patients. Until now, in BC, only three alterations have been reported to provide a clinical benefit in prospective randomized trials: *HER2* amplification, *PIK3CA* mutations, and *BRCA1* and *BRCA2* germline mutations.

Lastly, beside the gene mutation and amplification profiles of BC, MSI status is also gaining in interest as a biomarker for immunotherapy [28]. Many clinical trials are actually evaluating the interest of combining immunotherapy with either endocrine therapy in HR+ BC, or anti-HER2 therapies in HER2+ BC [29].

The present study is a proof of concept, retrospective study of integrated NGS characterization of HER2 low tumors, evaluating the feasibility of using NGS for determination of the *ERBB2* gene amplification in BC tumor specimens that have already undergone routine IHC for ER, PR, Ki67, and HER2 expression. This study also focuses on the potency of NGS analysis to identify simultaneously additional actionable molecular targets as well as MSI. The routine (Figure 1A) and the experimental (Figure 1B) workflows are illustrated in Figure 1.

## 2. Material and Methods

### 2.1. Tumor Specimens

Thirty-one FFPE BC tumor specimens were selected from the institutional tumor bank of the Lorraine Cancer Institute (authorization nr. DC-2013-1910 from the French Ministry of Research). According to the French regulation (art L-1211-2 of the Public Health Code, law nr. 2004-800, 6 August 2004). 

### 2.2. Routine Diagnostic Biomarkers Analyses

The tumors were fixed in 4% buffered aqueous formaldehyde for at least 12 h (biopsies) or 24 h (surgical specimens), then paraffin-embedded. Five µm slides were then prepared, and BC tumors specimens were stained by hematoxylin and eosin (CoverStainer Dako, Agilent Technologies, Les Ulis, France). CK19 (clone RCK 108, Agilent Technologies), estrogen receptor (ER, clone SP1, Ventana Roche Diagnostics, Meylan, France), progesterone receptor (PR, clone 1E2, Ventana Roche Diagnostics), HER2 (clone 4B5, Ventana Roche Diagnostics), and Ki-67 (clone MIB-1, Dako, Agilent Technologies) expression were determined using immunohistochemistry. All IHC assays were automated (Benchmark Ultra Ventana, Roche Diagnostics or Dako Omnis, Agilent Technologies) and processed according to the manufacturer’s recommendations.

The routine diagnostic analyses for BC were performed as recommended by the ASCO-CAP [30] and the French Unicancer GEFPICS group «Groupe d’Etude des Facteurs Pronostiques Immunohistochimiques dans le Cancer du Sein» [31].

The histopathological grading of the BC was performed according to the classification of the Scarff–Bloom–Richardson modified score. According to the recommendations of the GEFPICS [31], the scoring criteria that were used exactly followed the international recommendations. The threshold for positivity of ER and PR expression was set at 10% of labelled cells. Ki-67 expression was expressed as a percentage of the labelled cells. HER2-positive status was defined on the basis of complete intense membrane staining of more than 10% of the tumor cells (3+) or intermediate IHC overexpression (2+), defined as weak–moderate membrane staining in >10% tumor cells or intense membrane staining in <10% tumor cells with gene amplification (ISH+). IHC2+ tumors were analyzed using the IVD HER2 Dual ISH DNA Assay (D-DISH) (Ventana, Roche Diagnostics) to detect the amplification of the *ERBB2* gene using chromogenic probes that bind to the *ERBB2* gene. As a reference, labeling of chromosome 17 centromere (Chr17) was performed. According to the ASCO/CAP and GEFPICS recommendations, the amplification status is defined as amplified if the *ERBB2*/Chr17 ratio > 2.0, and as non-amplified if the HER2/Chr17 ratio < 2.0. If the *ERBB2*/Chr17 ratio falls between 1.8 to 2.2, an additional 20 nuclei should be enumerated, and the ratio re-calculated on the basis of all 40 nuclei.

### 2.3. Next Generation Sequencing

FFPE tissues were macrodissected after hematoxylin-eosin slide examination and after the tumor content was assessed by an experienced pathologist. DNA was then extracted using the QIAamp GeneRead DNA FFPE tissue kit (Qiagen, Courtaboeuf, France), and DNA concentrations were measured using the Qubit dsDNA HS assay kit and Qubit 3.0 Fluorometer instrument (ThermoFisher Scientific, Illkirch, France) according to the manufacturer’s recommendations. DNA quality was evaluated by electrophoretic profiles analysis using the high sensitivity large fragment analysis kit (Fragment Analyzer, Agilent Technologies). Genomic DNA Quality Number (GQN) score was calculated for each sample, corresponding to the proportion of DNA fragments with a size greater than the threshold set at 300 bp. Highly degraded DNA have a GQN close to 0, indicating that none of the fragments exceeds the threshold. Conversely, a good quality DNA have a GQN close to 10, with 100% of the DNA fragments being above the threshold value. However, considering the preciousness of the samples, no threshold was applied for GQN, and GQN values were considered during the post-analytical procedure, among all other quality criteria, to validate the results.

NGS libraries were prepared from 100 ng of extracted tumour DNA using a custom-designed, capture-based “Solid Tumour Solution” kit (Sophia Genetics, Geneva, Switzerland) that covers the regions of clinical interest of 51 cancer-associated genes as described elsewhere [32], among which are BC molecular target genes such as *ESR1*, *PIK3CA*, *AKT1*, *ERBB2*, *TP53*, *BRCA1*, and *BRCA2*. Targeted sequencing was performed using the MiSeq sequencer (Illumina). Generated raw NGS data were analyzed using the Sophia DDM software (v.5.10.37) (Sophia Genetics). Briefly, the bioinformatics pipeline (v. 5.5.79) consists of an alignment of the fastq files to generate bam files (hg19 reference genome), followed by a variant calling for the determination of SNV and indels. All results are finally available in the Sophia DDM software as retained variants and low-confidence variants. Quality control limits were required for each sample tested by NGS: >1 M reads/sample, >95% mapped reads, >70% on target, >85% at 500× depth. All pathogenic (class 5) and likely pathogenic (class 4) mutations detected among the 51 gene panel were reported. Copy Number Variation (CNV) was also calculated using CNV module from Sophia DDM software working in the gene amplification mode, i.e., only whole-gene duplications with a sufficiently large average copy number were reported. The threshold for gene amplification was set at 3.25, as recommended by the supplier of Sophia DDM (Sophia Genetics). Deletions and CNVs affecting only parts of genes were ignored. The gene amplification analysis included 24 genes (*NRAS*, *ALK*, *SF3B1*, *RAF1*, *PIK3CA*, *FGFR3*, *PDGFRA*, *KIT*, *FBXW7*, *TERT*, *ROS1*, *EGFR*, *MET*, *BRAF*, *FGFR1*, *CDKN2A*, *RET*, *FGFR2*, *HRAS*, *MYOD1*, *KRAS*, *CDK4*, *TP53*, *ERBB2)*. The coverage levels used for gene amplification detection included a linear GC correction deduced from all of the regions included in the panel. MSI was determined using an algorithm module provided by Sophia DDM software based on reads analysis at 6 unique MS regions, for which 3 were split into forward (FWD) and reverse (REV) strands (BAT25_FWD, BAT25_REV, BAT26_REV, CAT25_REV, NR-21_FWD, NR-21_REV, NR-22_FWD, NR-22_REV, and NR-27_REV), as previously validated in FFPE tissues from endometrial and colon cancers [32].

### 2.4. Statistical Analysis

The sample size was calculated consistently with the recommendations of the French Committee for Accreditation (COFRAC Comité Français d’accréditation) in its technical guidelines for accreditation and validation of methods in medical biology (Guide technique d’accréditation de validation des méthodes de biologie médicale SH GTA 04—Revision 02 https://tools.cofrac.fr/documentation/sh-gta-04 (accessed on 15 November 2022)). The sample size of this study was justified considering the Cohen’s effect size using the pairwise and unpaired comparison test. In such conditions, sample groups (HER2+ vs. HER2-low and HER2+ versus HER2−) were compared to identify a significant standardized difference. The ranges for Cohen’s effect size were, respectively, 0.10 < d < 0.30 (small effect), 0.30 < d < 0.50 (moderate effect), and d ≥ 0.50 (large effect). Data were compared using the non-parametric Wilcoxon rank sum test. Statistical analyses were performed using R-studio software (version 2022.07.2+576) with rstatix and Wilcox test () functions. A *p*-value < 0.05 was used as a significativity limit.

## 3. Results

Thirty-one FFPE BC tumor specimens were selected from the tumor bank based on IHC and ISH results of HER2 status obtained during routine first line BC testing, together with hormone receptors (ER and PR) and Ki67 IHC analyses.

Among the 31 tumors, 5 were classified as grade 1 (SBR-EE classification), 17 as grade 2, and 9 as grade 3. Most of the tumors were hormone receptor positive (29/31), defined as positive for ER or PR expression with 29/31 ER+ and 23/31 PR+. All tumors had ER/PR expression above 10%, therefore making no difference between ASCO/CAP and GEFFPICS classification.

HER2 IHC results show that 10/31 tumors were IHC 0, 5 IHC1+, 9 IHC2+, and 7 IHC3+. The 9 HER2 IHC2+ tumors were further analyzed using ISH, and *HER2* amplification was observed in 4/9 tumors (44%). In total, the analyzed BC population consisted of 10 HER2 negative, 10 HER2-low, and 11 HER2 positive tumors. HER2-negative and HER2-low tumors were mostly grade 2 tumors (6/10 in HER2-negative and 7/10 in HER2-low), as opposed to HER2-positive tumors that were mostly composed of grade 3 tumors (7/11).

**Figure 2:**
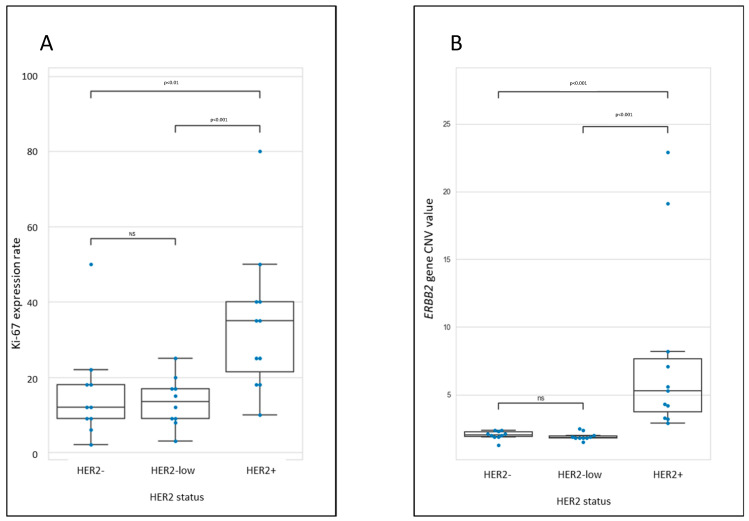
Distribution of Ki67 expression rate (**A**) and *ERBB2* gene copy number variation (CNV) values (**B**) in the 31 HER2-negative, HER2-low and HER2-positive samples. (ns: non significant difference).

KI67 labeling ranged from 2 to 80%, and was significantly higher (*p* < 0.001) in HER2-positive (IHC3+ or IHC2+ and ISH+) than in HER2-negative (IHC0) and HER2-low (IHC1+ or IHC2+, ISH−) BC, with mean values of 34.2 (SD = 19.2), 15.8 (SD = 13.4) and 13.5 (SD = 6.5), respectively (Figure 2A).

All analyzed macrodissected samples had tumor cell content exceeding 10% (range 15–80%) after examination of HE-stained slides by an expert pathologist. No difference was observed between the different groups.

Using NGS, CNV values for the *ERBB2* gene were significantly different (*p* < 0.001) between HER2-positive, either HER2-low or HER2-negative tumors with mean values of 7.8 (SD = 6.8), 1.9 (SD = 0.3), and 2.0 (SD = 0.3), respectively (Figure 2B). With a cutoff value for CNV set at 3.25, HER2 amplification status was consistent between ISH and NGS in all but one sample (sample #24). This sample has discordant results with a CNV value of 2.9, while having a weak positive HER2/Chr17 ISH ratio of 2.2 and a tumor cell content of 40%, close to the threshold values. In addition, the DNA extract from this sample had a low GQN value of 1.5, illustrating a low quality DNA that could explain the outlying. As a whole, the results for HER2 status were achieved following the experimental workflow (IHC combined with NGS produced results that were consistent with those achieved using the reference workflow (IHC combined with ISH) in 30/31 cases i.e., 96.8%).

Considering 21 samples for comparison of HER2+ (N = 11) and HER2− (N = 10) or HER2+ (N = 11) and HER2-low (N = 10), evidenced in each case was an effect size of 0.85 (large effect), therefore validating the sample size.

Additional gene amplifications were simultaneously detected in 19% (6/31) of the cases, either in HER2-negative tumors (2 cases with *FGFR1* amplifications and 2 with *MYOD1* amplifications) and in HER2-positive tumors (1 case with *FGFR1* amplification, 1 with *PIK3CA* amplification and 1 with *ROS1*amplification) (Supplementary Figure S1).

Additional pathogenic and likely pathogenic gene mutations were detected among the 51 gene panel in 68% (21/31) of the cases with 12 *PIK3CA*, 6 *TP53*, 2 *AKT1*, and 4 *BRCA2* mutations.

Further, one case of MSI status was detected in a HER2-negative, ISH unamplified, HR-positive tumor bearing a *PIK3CA* gene mutation (c.1624G > A, p.(Glu542Lys)).

All data are summarized in Table 2.

## 4. Discussion

Based on the expression of hormone receptors (HR) and HER2, BC used to be classified into four intrinsic subtypes: luminal A (HR+ and HER2−), luminal B (HR+ and HER2+), HER2-enriched (HR− and HER2+), and triple-negative BC (TNBC, HR− and HER2−) [33], among which TNBC is generally considered to have the worst prognosis.

The identification of HER2-low BC has recently reached great clinical significance [34]. Usually reported as HER2-negative and classified as TNBC or luminal-like in clinical practice, HER2-low BC is a heterogeneous disease and shows distinct molecular profile and prognosis compared with tumors negative for HER2 by IHC [35].

However, since negative results have been reported with the addition of trastuzumab or pertuzumab to chemotherapy in patients with HER2-low BC [13], HER2-low BC were not eligible for anti-HER2 monoclonal antibodies.

The DESTINY-Breast04 phase 3 trial revealed the efficacy of T-DXd in HER2-low metastatic breast cancers previously treated with one or two lines of chemotherapy. As compared to physician-selected chemotherapy, T-DXd significant increased PFS and OS in the HR+ cohort [36].

In the DAISY trial [26], even very low levels of HER2 expression appeared to drive T-DXd cellular uptake and other mechanisms independent of HER2, and were suggested to be also partially involved in the efficacy of T-DXd.

The identification of HER2-low status is therefore becoming crucial in the management of BC, but remains challenging due to methodological and analytical variables that might influence the sensitivity and reproducibility of HER2 IHC and ISH routine testing. Additionally, the heterogeneity of HER2 expression and/or amplification should be considered for improving HER2-low identification. Since HER2 heterogeneity could be seen in both HER2 gene copy number and/or protein expression, guidelines have been updated by the ASCO/CAP [4], but the consensus to define HER2 heterogeneity for IHC is still lacking [37,38]. This sounds particularly important since HER2 IHC heterogeneity has been reported to be more frequent in the HER2-low samples [39] and might contribute to variability of HER2-low interpretation, and consequently influence the response to ADCs [40].

The development of NGS technologies has raised hopes for precision medicine treatment strategies in breast cancer (BC) and triple-negative breast cancer. Multiple additional molecular targets can now be screened in BC, as illustrated by the ESCAT classification [27]. Alterations that have been recently added in international guidelines [41] include the *ESR1* gene mutations and those that still need confirmatory prospective studies, but for which evidence of efficacy is available, including the *PIK3CA*, *AKT1*, *HER2*, *TP53*, *BRCA1*, and *BRCA2* gene mutations that were included in the custom capture-based 51-gene NGS panel used in this study.

Besides mutations, the identification of gene amplifications is of increasing interest for diagnostic, pronostic, and theranostic purposes in BC. This is the case for some amplifications that were identified in our study. *MYOD1* amplification was reported to be of pronostic interest in BC, with the greatest prevalence in breast invasive lobular carcinoma as well as *ROS1* amplification [40]. *FGFR1* amplification was reported to be associated with bad prognosis in hormone-positive BC and resistance to hormonal monotherapy, or in combination with palbociclib. Resistances can be reverted with triple ER, CDK4/6, and FGFR1 blockade [42]. *PIK3CA* mutations and amplifications have been reported in approximately 10% of HR-positive and HER2-negative primary BC and among patients with *PIK3CA*-mutated BC; those with amplified *PIK3CA* have worse outcomes [43].

Finally, MSI status can serve as a biomarker to initiate immunotherapy in TNBC and also in HER2-negative HR-positive metastatic BC [28]. In this context, although MSI status is detected in a low proportion of BC [44], its simultaneous determination using NGS can be valuable to allow the initiation of immunotherapy, alone or in combination with endocrine therapy in clinical trials upon molecular board decision [29].

Facing the availability of tumor tissue in limited quantity, which is very often already consumed by multiple IHC and ISH procedures, molecular pathologists are often confronted to choose between rapid PCR-based diagnostic with short time-to-results (TTR) delay (1–3 days in routine practice) limited to a relatively small number of target genes. In such a context, extensive NGS with large gene panels in the several hundreds can prove very helpful, although generating a longer TTR (1–3 weeks in routine practice). While optimizing the use of small biopsies, using NGS could allow for the selection of innovative therapeutic options based on molecular tumor board decision guided by the ESCAT scale [27], and/or to propose to the patient that they be included in a clinical trial [45].

In the present study we show that, following first-line IHC routine analysis alone, NGS would provide biomarkers and identify multiple targets as well as MSI status, which is helpful in HER2-low and TNBC. Using NGS can spare biological material and time, and can prove useful for tumor stratification upon molecular board. This study warrants further confirmation in a larger patient sample population.

## Figures and Tables

**Table 1:** ESCAT ranking of molecular classification.

Tier I	Clinically relevant	A: survival benefit in prospective, randomised trials B: clinical benefit in prospective, single-arm trials C: clinical benefit in basket trials
Tier II	Potentially clinically relevant	A: clinical benefit in retrospective trials B: increased responsiveness and outcome in prospective trials
Tier III	Potentially clinically relevant	A: clinical benefit in other tumor entities B: biomarker is located in the same gene/pathway as Tier I-IIIA targets
Tier IV	Evidence from preclinical studies	A: improved drug sensitivity in in-vitro or in-vivo models B: actionability shown in in silico models
Tier V	co-targeting approaches	Improved objective response in prospective trials
Tier X	No evidence	Biomarkers are not actionable

**Table 2:** Histological and molecular characteristics of the tumors: Breat cancers specimens were routinely characterized using Immunohistochemistry (IHC) and In situ hybridization (ISH) for hormonal status, defined from estrogen receptor (ER) and Progesterone Receptor (PR) expression, Ki67 labeling index, HER2 expression and HER2 gene amplification. The tumor cell content was determined by expert pathologist to validate the macrodissection area. Next Generation Sequencing (NGS) was further used in this study for determination of gene amplifications defined by Copy number Variation (CNV ) > 3.25, gene mutations analysis with variant allelic fraction (VAF) determination. Only pathogenic (class 5) and likely pathogenic (class 4) mutations were recorded. Microsatellite instability (MSI) status defined as microsatellite instable (MSI) or microsatellite stable (MSS).

	Grade	Hormone Receptors			Ki67	Tumor Cells Content	HER2/ERBB2			Additional Gene Amplifications		Additional Gene Mutations					MSI
Sample	SBR-EE	ER	PR	HR Status	(%)	(%)	IHC/ISH	HER2 Status	CNV *ERBB2*	Gene	CNV	Gene	c.	P.	VAF	Pathogenicity	MSS/MSI
1	2	ER+	PR+	HR+	12	70	IHC 0	HER2−	2.4			*PIK3CA*	c.1633G>A	p.(Glu545Lys)	34%	5	MSS
2	3	ER+	PR-	HR+	50	70	IHC 0	HER2−	2.3			*TP53*	c.267_269delinsTT	p.(Trp91Glyfs*32)	39%	4	MSS
3	2	ER+	PR+	HR+	9	80	IHC 0	HER2−	2.1								MSS
4	2	ER+	PR+	HR+	18	60	IHC 0	HER2−	1.3			*PIK3CA*	c.1624G>A	p.(Glu542Lys)	31%	5	MSI
5	1	ER+	PR+	HR+	6	80	IHC 0	HER2−	2.4	*MYOD1*	3.4	*PIK3CA*	c.1633G>A	p.(Glu545Lys)	36%	5	MSS
6	2	ER+	PR+	HR+	18	70	IHC 0	HER2−	2.1			*TP53*	c.548C>G	p.(Ser183*)	6%	4	MSS
												*AKT1*	c.49G>A	p.(Glu17Lys)	59%	5	
7	1	ER+	PR+	HR+	9	70	IHC 0	HER2−	1.9			*PIK3CA*	c.3140A>G	p.(His1047Arg)	26%	5	MSS
												*AKT1*	c.49G>A	p.(Glu17Lys)	32%	5	
8	2	ER+	PR+	HR+	22	60	IHC 0	HER2−	1.9								MSS
9	2	ER+	PR-	HR+	2	20	IHC 0	HER2−	2.0			*PIK3CA*	c.3140A>G	p.(His1047Arg)	5%	5	MSS
10	1	ER+	PR+	HR+	12	15	IHC 0	HER2−	2.0								MSS
11	1	ER+	PR-	HR+	17	60	IHC1+	HER2− (HER2 low)	1.8	*FGFR1*	5.8						MSS
12	2	ER+	PR+	HR+	20	70	IHC1+	HER2− (HER2 low)	1.9			*PIK3CA*	c.1633G>A	p.(Glu545Lys)	27%	5	MSS
13	2	ER+	PR+	HR+	12	70	IHC1+	HER2− (HER2 low)	1.8								MSS
14	2	ER+	PR+	HR+	9	30	IHC1+	HER2− (HER2 low)	1.8			*PIK3CA*	c.3140A>G	p.(His1047Arg)	2%	5	MSS
15	1	ER+	PR+	HR+	3	30	IHC1+	HER2− (HER2 low)	1.9			*RET*	c.2372A>T	p.(Tyr791Phe)	49%	4	MSS
16	2	ER+	PR+	HR+	15	80	IHC2+ ISH-	HER2− (HER2 low)	1.8								MSS
17	2	ER+	PR-	HR+	8	70	IHC2+ ISH-	HER2− (HER2 low)	2.4			*BRCA2*	c.9376C>T	p.(Gln3126*)	35%	5	MSS
18	2	ER+	PR+	HR+	17	80	IHC2+ ISH-	HER2− (HER2 low)	1.5								MSS
19	3	ER+	PR+	HR+	25	75	IHC2+ ISH-	HER2− (HER2 low)	2.5								MSS
20	2	ER+	PR+	HR+	9	60	IHC2+ ISH-	HER2− (HER2 low)	2.0	*MYOD1*	4.2	*BRCA2*	c.3830del	p.(Asn1277Ilefs*7)	49%	5	MSS
21	3	ER+	PR+	HR+	35	80	IHC2+ ISH+	HER2+	3.3			*PIK3CA*	c.1624G>A	p.(Glu542Lys)	8%	5	MSS
22	3	ER+	PR+	HR+	50	70	IHC2+ ISH+	HER2+	4.2			*PIK3CA*	c.1633G>A	p.(Glu545Lys)	44%	5	MSS
												*BRCA2*	c.5702_5703del	p.(Glu1901Glyfs*5)	40%	5	
23	3	ER-	PR-	HR-	40	30	IHC2+ ISH+	HER2+	3.3			*TP53*	c.614A>G	p.(Tyr205Cys)	7%	4	MSS
												*PIK3CA*	c.3140A>G	p.(His1047Arg)	9%	5	
24	2	ER+	PR+	HR+	10	40	IHC2+ ISH+	HER2+	2.9			*PIK3CA*	c.3140A>G	p.(His1047Arg)	24%	5	MSS
25	2	ER+	PR+	HR+	18	40	IHC3+	HER2+	5.6								MSS
26	2	ER+	PR+	HR+	18	70	IHC3+	HER2+	8.2			*TP53*	c.637C>T	p.(Arg213*)	30%	4	MSS
27	3	ER+	PR+	HR+	35	70	IHC3+	HER2+	22.9			*TP53*	c.832C>T	p.(Pro278Ser)	37%	4	MSS
28	2	ER+	PR+	HR+	25	30	IHC3+	HER2+	7.1	*PIK3CA ROS1*	5.5 6.2	*BRCA2*	c.5073dup	p.(Trp1692Metfs*3)	13%	5	MSS
29	3	ER-	PR-	HR-	80	60	IHC3+	HER2+	5.3			*TP53*	c.584T>C	p.(Ile195Thr)	17%	4	MSS
30	3	ER+	PR-	HR+	25	60	IHC3+	HER2+	4.3	*FGFR1*	4.4	*SF3B1*	c.2098A>G	p.(Lys700Glu)	29%	4	MSS
												*PIK3CA*	c.3140A>G	p.(His1047Arg)	25%	4	
31	3	ER+	PR-	HR+	40	25	IHC3+	HER2+	19.1	*FGFR1*	4.4						MSS

## Data Availability

The data presented in this study are available on reasonable request from the corresponding author. The data are not publicly available due to ethical reasons.

## Supplementary Materials

The following supporting information can be downloaded at: https://www.mdpi.com/article/10.3390/biomedicines11123164/s1.
