# Supplementary material for: Integrated Molecular Characterization of HER2-Low Breast Cancer Using Next Generation Sequencing (NGS)
Source: Biomedicines. 2023 Nov 28;11(12):3164. doi: 10.3390/biomedicines11123164 (PMC10740754; doi:10.3390/biomedicines11123164)
Supplement: Supplementary file 1 [file biomedicines-11-03164-s001.zip › suppl table 1.pdf]

Table S1. Quality control data of the tumor specimens and NGS analyses. No threshold was applied for GQN[a]. The quality control limit for NGS were > 1M reads / sample[b], > 95% mapped reads[c], > 70% on target[d], > 85% at 500X depth[e]

Sample	GQN [a]	N reads / sample [b]	mapped reads (%) [c]	on target (%) [d]	depth 500x (%) [e]
**1**	3.0	1,339,556	97.3	78.87	99.90
**2**	3.1	1,065,970	98.03	80.53	99,11
**3**	2.9	2,100,262	97.98	79.02	100.00
**4**	3.4	1,434,004	98.21	80.66	99.85
**5**	1.6	1,812,518	98.39	81.36	100.00
**6**	4.2	1,865264	98.25	79,62	95.53
**7**	2.9	1,971,914	98.67	81.60	100.00
**8**	2.1	1,468,368	98.61	82.68	100.00
**9**	3.5	1,523,612	98.81	81.73	99.99
**10**	3.6	2,92375	98.80	81.92	100.00
**11**	3.9	2,403,544	98.82	81.41	100.00
**12**	3.9	1,693,384	98.76	82.86	100.00
**13**	3.6	1,373,600	98.87	80.43	100.00
**14**	3.2	1,989,142	98.82	80.46	100.00
**15**	4.4	1,401,394	98.99	82.28	98.88
**16**	3.9	1,670,492	98.73	81.26	100.00
**17**	3.3	1,486,460	97.36	82.90	97.48
**18**	1.8	1,205,424	96.41	81.44	97.18
**19**	2.6	1,094,540	97.64	82.07	98.39
**20**	4.6	1,454,484	97.44	82.98	98.45
**21**	3.2	1,200,628	97.29	83.50	99.03
**22**	5,0	1,196,106	97.64	82.05	95.48
**23**	2.6	1,287,744	98.91	81.32	100.00
**24**	1.5	1,452,372	98.78	80.96	97.93
**25**	4.9	2,202,750	99.04	83.15	100.00
**26**	4.0	2,606,896	98.99	81.61	100.00
**27**	2.4	5,117,360	99.06	80.88	100.00
**28**	2.0	2,214,702	98.58	82.60	100.00
**29**	4.6	2,335,432	98.90	80.96	100.00
**30**	5.3	2,358,204	98.84	80.22	100.00
**31**	5.1	1,873,700	98.93	84.82	99.79
