# Supplementary figures and images for: Integrated Molecular Characterization of HER2-Low Breast Cancer Using Next Generation Sequencing (NGS)
Source: Biomedicines. 2023 Nov 28;11(12):3164. doi: 10.3390/biomedicines11123164 (PMC10740754; doi:10.3390/biomedicines11123164)

Figure S1. Hitmap of CNV values of the 24 genes analyzed for CNV in the 31 tumor samples

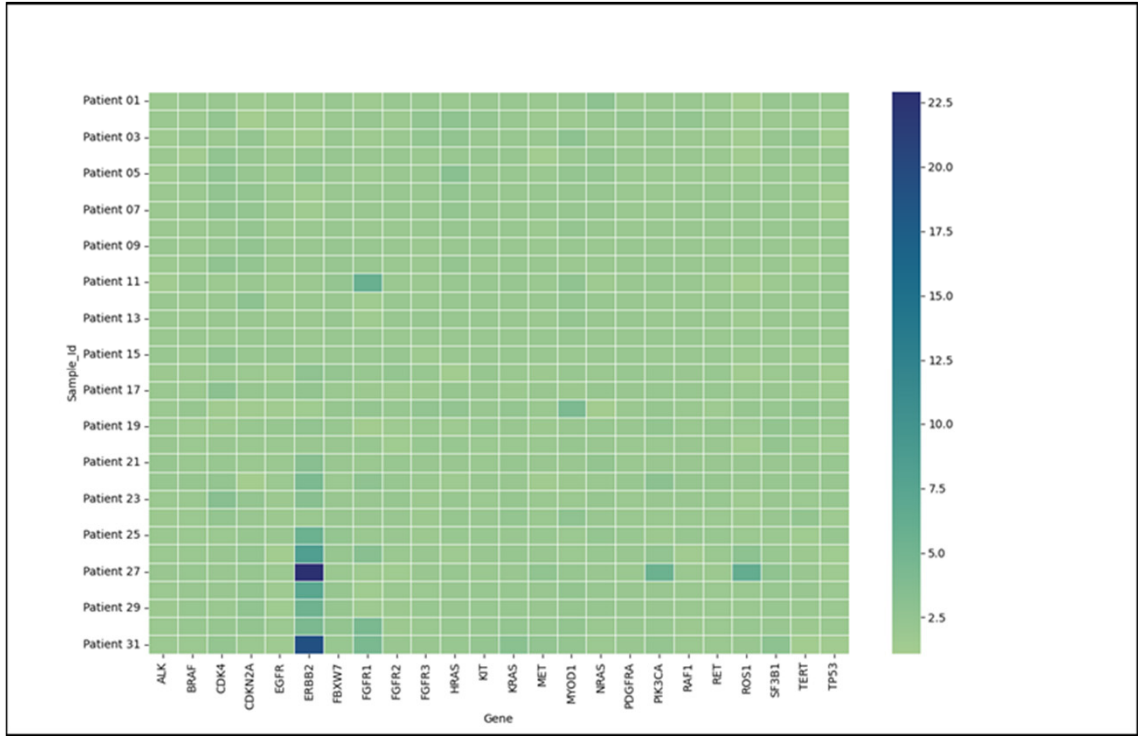

Supplement: Supplementary file 1 [file biomedicines-11-03164-s001.zip › suppl fig 1.pdf]
